# Editorial: Role of Health Economic Data in Policy Making and Reimbursement of New Medical Technologies

**DOI:** 10.3389/fphar.2017.00662

**Published:** 2017-09-21

**Authors:** Mihajlo Jakovljevic, Tetsuji Yamada

**Affiliations:** ^1^Global Health Economics and Policy, Faculty of Medical Sciences, University of Kragujevac Kragujevac, Serbia; ^2^Center for Health Trends and Forecasts, Institute for Health Metrics and Evaluation, University of Washington Seattle, WA, United States; ^3^Economics Department, Rutgers University, State University of New Jersey New Brunswick, NJ, United States

**Keywords:** health economics, decision making, resource allocation, health expenditure, reimbursement, medical technology

This Research Topic was created with a mission to tackle the core challenges for the provision of new medical technologies across the globe considering increasing prices and finite financial resources (Malmström et al., 2013; Permanand and Pedersen, 2015). A key area certainly includes anti-cancer drugs where prices have increased 10-fold in recent years (Kelly and Smith, 2014) leading to concerns with affordability for both health services and patients (Ghinea et al., 2015; Tefferi et al., 2015). The objective was to reveal some of the hidden underlying causes of unequal access to the medicines as well as the growing proportion of out-of-pocket health spending in many world regions (Global Burden of Disease Health Financing Collaborator Network, 2017). In line with the joint efforts of the editors and authors we received an exceptionally high response worldwide. The topic attracted a total of 36 self-standing research submissions out of which 31 ultimately passed external peer review and got published. Base affiliations of the authors spread across academia, pharmaceutical and medical device industry, governmental authorities and clinical medicine. Their home institutions were situated in 15 different countries inclusive of Japan, Israel, Russia, USA, Germany, Italy, Netherlands, Austria, Spain (Basque), Malta, Serbia, Poland, Bulgaria, Hungary and Malaysia.

In published health economics literature, there is straightforward evidence that accelerated growth of health spending began in the 1960s exceeding the historical 4% GDP threshold (Jakovljevic and Ogura, 2016). This phenomenon was noticed early on in mature market economies led by the US, and during the following decades spread to many global regions (Getzen, 2000). Health policy makers became increasingly exposed to new harsh challenges in the uneasy task to provide universal health coverage and decent equity of access to medical services. Among the most prominent demand-side issues are population aging (Murata et al., 2010), rise of non-communicable diseases (Jakovljevic and Milovanovic, 2015), and growing patient expectations. Supply-side causes include improvements in societal welfare (Yamada et al., 1992) and living standards, technological innovation in medicine, and continuing rapid urbanization in developing world regions. Experience with implementation of insurance-based risk sharing agreements which aim to facilitate access to new medicine varies substantially (Adamski et al., 2010; Ferrario and Kanavos, 2013; Ferrario et al., 2017). There are growing measures to enhance the prescribing of low cost generics and biosimilars across Europe and other countries without compromising care as the savings can be considerable and used to fund new technologies (Cameron et al., 2012; Simoens, 2012; Vogler, 2012; Godman et al., 2014). Published studies have shown that prices of good quality generics can as low as 2% of prepatent loss prices (Woerkom et al., 2012). Also, the considerable build-up of workforce capacities and strengthening of primary care and hospital networks contributed to the “supplier induced demand” phenomenon (Richardson and Peacock, 2006).

There is straightforward historical evidence of long term growth in pharmaceutical and overall health spending both in absolute and GDP % terms worldwide (Dieleman et al., 2017). The accumulated constraints resulting from rising costs of care were felt in many areas of clinical medicine even among the richest societies (Kotlikoff and Hagist, 2005). Examples of expensive and hardly affordable novel therapeutic areas are orphan drugs indicated to treat rare diseases (Cohen and Felix, 2014; Taruscio et al., 2015) and targeted biologicals used in autoimmune disorders and cancer; new cancer drugs often with limited health gain (Kantarjian et al., 2013; Wild et al., 2016). Frequently denied access to even essential generic pharmaceuticals (Jakovljevic et al., 2014) is still taking place, in rural and suburban areas of certain countries (e.g., Japan). These difficulties are worsened by the lack of evidence-based resource allocation strategies and less sustainable financing strategies (Jakovljevic et al., 2016).

Core goals of the Editors of this collection of articles were to cover a growing gap between the medical technology innovation, its dissemination, and cost containment issues. The European Commission has estimated that there is almost 36% room for efficiency gains and costs reductions in most contemporary European health systems (COST Action, 2016). So-called “emerging costs for healthcare” have an estimated growth of almost €1,400 billion annually EU wide. Similar issues were clearly recognized in other major global health care markets such as USA (Anderson et al., 2005) and Japan (Ogura and Jakovljevic, 2014) among the mature ones, and the BRICs led by People's Republic of China among the emerging ones (Jakovljevic, 2015). In addition, situation in China is hampered by the hospitals' and physicians' need for profits for their survival made from drug procurement which leads to the high use of injections and infusions (Reynolds and McKee, 2011; Yang et al., 2013; Zeng et al., 2014). There is an ongoing public debate about the effective introduction and spreading of value based medicine concepts, and introduction of cost-effectiveness criteria into official policy making in most world regions. Since the pioneering moves by Australia (Parker and Guthrie, 1993) and Canada (Grosse, 2008) back in early 1990s, in many countries these efforts were rather slow and less successful. To a large extent the solution was found in health technology assessment (HTA) procedures and establishment of a strong network of national HTA Agencies in North America, Europe and Asia (Banta and Jonsson, 2009). However, a thorough search through the health economics literature testifies that authorities and experts alike are shifting their focus of interest toward other possible strategies to deliver cost-effective health care (Neumann, 2005). This is a notable challenge in low- and middle-income world regions (Stafinski et al., 2011) and even in some high-income OECD economies where HTA did not grasp its roots inside official policy making on resource allocation in health care (Perry et al., 1997). Particular challenge in low- and middle-income countries include the fact that medicines may account for up to 70% of total health care expenditure, much of which is currently out-of-pocket, although starting to change with Namibia and South Africa striving for universal access (Cameron et al., 2009).

One group of contributions to our topic referred to the Eastern European health systems of Hungary, Poland, Bulgaria, Serbia and Bosnia. The University of Debrecen, Hungary, brought attention to the relationship between statin prescription and socioeconomic deprivation of patients (Boruzs et al.). However, restrictions in countries such as Lithuania where only patented statins are available eased with generic availability enhancing utilization (Garuolienė et al., 2016). On the other hands some authors have questioned whether increasing statin utilization reduces cardiovascular diseases (Vancheri et al., 2016). The Medical University of Silesia in Poland conducted several studies covering the areas: health promotion development in spa treatment (Woźniak-Holecka et al.), perspectives of use of social media in pharmaceutical marketing (Syrkiewicz-Świtała et al.), systemic changes in efficiency of Polish primary health care (Holecki et al.), and clustering policy effects within their national health system (Romaniuk et al.). The Medical University of Plovdiv, Bulgaria, acting as one of the European centers of excellence in rare diseases and orphan medicines, published a piece on importance of the socio-economic burden as a decision-making criterion (Iskrov et al.). University of Belgrade and Institute of Public Health in Serbia jointly presented insight into the socio-economic inequalities, out-of-pocket payments in large national consumers' satisfaction survey samples (Vojvodic et al.). The Military Medical Academy in Belgrade contributed with a study on pharmaceutical expenditure and burden of non-communicable diseases in Serbia (Kovacevic et al.). This was complemented by findings on contribution of health workforce to the structure of health spending (Jakovljevic and Varjacic) coming from the University of Kragujevac, Serbia. Other reported trials from this country refer to hepatitis treatment among former addicts (Jovanovic et al.), socioeconomic factors associated with psychoactive substance abuse among the adolescents (Janicijevic et al.), and tobacco use patterns (Vasiljevic et al.). A variety of clinical entities ranging from dentistry (Djordjevic et al.) to the gynecological conditions and fertility (Djukic et al.), were processed as well due to their relevance for the national health system financing and medical service provision. Nation-wide health surveys in the country were used as a ground for research on citizen satisfaction with health sector (Mihailovic et al.) and self-assessed health and socioeconomic inequalities (Radevic et al.). A prominent piece providing a big picture on former Yugoslavia's health systems referred to length of hospital stay and bed occupancy rates (Cvetkovic et al.). Ultimately Western Balkan/former Yugoslavia—related research was concluded with a critical appraisal of Bosnia's medicines reimbursement list (Mujkic and Marinkovic).

Broader perspectives on Pan-European pharmaceutical spending brought in another contribution reflecting East-West split in expenditure evolution patterns inside EU (Jakovljevic et al.). There is a diversity of contributions provided by some academic centers based in Western European EU-15 countries. Here we witness a paper on alcohol beverage taxation and government revenues in European WHO Region brought to us by Vienna Medical University, Austria and Federal Research Institute for Public Health Organization and Information (CNIIOIZ) Moscow, Russia (Jakovljevic et al.). Dutch health economists delivered several prominent studies including ones on stratified medicine (Fugel et al.), barriers to market access of biosimilar monoclonal antibodies in European Union (Moorkens et al.), and industrial landscape in pharmaceutical biotechnology (Moorkens et al.). We had as well a valuable commentary coming from Malta reflecting ration between health expenditure growth in leading G7 industrialized nations compared to the BRICs (Buttigieg et al.) referring to the original article published in Journal of Medical Economics back in 2016 (Jakovljevic, 2016). These findings were complemented by a piece on long term health spending in low- and middle-income countries co-authored contribution of Temple University, Philadelphia, USA (Jakovljevic and Getzen). Western European cluster of papers had conclusive remarks on observed and normative functions applied to addiction disorders coming from Almeria University, Spain (Cruz Rambaud et al.) and the review on life cycle of health technologies (Gutiérrez-Ibarluzea et al.) brought to us jointly by the Basque and German Offices for HTA and Italian Society of Clinical Pharmacy and Therapeutics.

Ultimately a number of contributions coming from vast Asian continent referred to the commentary on population aging in Japan (Jakovljevic) as depicted in the original article by Fukushima et al. (2016). A self-standing piece on children medical expenses in current Japanese legislation came to us by Hosei University, Tokyo (Sugahara). Remaining studies published in this series were related to causal connection between physical exercise and pharmacological treatment of mood disorders such as major depression submitted by the Wingate Institute, Israel (Netz). Island state of Malaysia and its University Teknologi Petronas has published a piece on long term modeling of health expenditures. Keeping in mind strong pace of economic development in South-East Asia, these projections might be very valuable and fill an important knowledge gap on emerging markets (Khan et al.).

## Author contributions

MJ and TY have jointly designed the research question, prepared the manuscript and revised it for important intellectual content.

### Conflict of interest statement

The authors declare that the research was conducted in the absence of any commercial or financial relationships that could be construed as a potential conflict of interest.
